# Formative Evaluation of an HIV Prevention App tailored for Latino Men Who Have Sex With Men: Acceptability and Usability Study

**DOI:** 10.2196/74208

**Published:** 2025-10-28

**Authors:** Valeria D Cantos, Humberto Posada-Orozco, Isabella Batina, Natalie Sanchez, Eric Rangel, Patrick S Sullivan, Andres Camacho-Gonzalez, Aaron J Siegler

**Affiliations:** 1 Division of Infectious Diseases, Department of Medicine, Emory University School of Medicine, 68 Armstrong St. SE. Suite 210, Atlanta, GA, 30303, United States, 1 404-368-2656; 2 Duke University School of Medicine, Durham, NC, United States; 3 Latino Linq, Atlanta, GA, United States; 4 Department of Epidemiology, Rollins School of Public Health, Emory University, Atlanta, GA, United States; 5 Division of Pediatric Infectious Diseases, Department of Pediatrics, Emory University School of Medicine, Atlanta, GA, United States; 6 Department of Behavioral, Social, and Health Education Sciences, Rollins School of Public Health, Emory University, Atlanta, GA, United States

**Keywords:** mobile health, mHealth, pre-exposure prophylaxis, PrEP, acceptability, usability, pilot, Latino, HIV

## Abstract

**Background:**

HIV incidence is increasing among Latino gay, bisexual, and other men who have sex with men (MSM) in the Atlanta metropolitan area. Mobile phone apps represent an innovative tool to promote pre-exposure prophylaxis (PrEP) use, HIV testing, and condom use.

**Objective:**

This study aimed to assess the acceptability and usability of Saludfindr, an Android-based HIV prevention app tailored to the needs of Latino MSM in the Atlanta area.

**Methods:**

We recruited adult Latino MSM to interact with the app for 4 months. Saludfindr included initial and periodic health assessments; provision of suggestions regarding PrEP, HIV testing, and condom use; in-app product ordering; customized motivational messages; a customized sexual health clinic list; and a “Contact Us” button. To assess acceptability, we measured use of each app feature, PrEP and HIV testing uptake, and participant ratings of the app’s usefulness. We assessed usability using the System Usability Scale.

**Results:**

We enrolled 31 participants; the median age was 27 (IQR 24.5-32) years, 97% (30/31) were cisgender men, 81% (25/31) identified as MSM, and 61% (19/31) used the app in Spanish. All participants completed the initial health screening, with 84% (26/31) and 77% (24/31) completing the 2- and 4-month health screenings, respectively. Of all participants, 52% (16/31) and 23% (7/31) ordered condoms and home HIV tests through the app at least once, respectively. During the study period, 71% (22/31) of the participants got tested for HIV, of whom 68% (15/22) accessed it through clinic-based HIV testing. Of the participants not on PrEP at baseline, 41% (7/17) initiated PrEP during the study, and all of them did so at one of the clinics listed on the app. Saludfindr reached a System Usability Scale score of 74.5/100 (excellent).

**Conclusions:**

Saludfindr was highly acceptable and usable among Latino MSM participants in the Atlanta area. In-app assistance to access PrEP and clinic-based HIV testing referrals was well received. Further efforts are needed to increase users’ self-efficacy with home HIV self-testing.

## Introduction

### Background

Between 2018 and 2022, HIV incidence in the United States decreased 12% due at least in part to an overall increase in pre-exposure prophylaxis (PrEP) use [1]. These advancements toward ending the HIV epidemic have not reached all racial and ethnic groups equally. Nationally, HIV incidence among Latino gay, bisexual, and other men who have sex with men (MSM) remained unchanged between 2018 and 2022 while decreasing 16% and 20% among Black and White MSM, respectively [2]. In 2022, Latino MSM ranked first in number of new HIV diagnoses among MSM, accounting for 36% of all new diagnoses among MSM [2].

The state of Georgia has the third highest rate of HIV diagnosis among Latina, Latino, or Latinx individuals in the United States, with 80% of cases occurring in the Atlanta metropolitan area [3]. Between 2010 and 2019, HIV diagnosis rates among Latino men in Georgia, 80% of them MSM, increased by 13% while decreasing among Black and White men (−12.3% and −21%, respectively) [4].

Despite comparable PrEP awareness among White, Black, and Latino MSM [2], significant inequities in PrEP uptake are observed [5]. The PrEP-to-need ratio (PnR), a measurement assessing PrEP use compared to HIV diagnoses in the same period for the same population or region, is widely used as an estimation of PrEP equity, with a lower PnR representing a higher unmet need [6]. In 2021, the PnR for Latina, Latino, or Latinx individuals was over 4 times lower compared to the PnR for White individuals (6.0 vs 25.9) [5].

Latino MSM face significant barriers to PrEP uptake in metropolitan Atlanta [7-9]. Some of these barriers, such as low perceived PrEP eligibility [7,10-12], intersectional stigma [7,13-15], and PrEP misconceptions [7,16], are also common among other racial and ethnic groups in the United States. However, many PrEP uptake barriers among Latino MSM are related to its limited accessibility [7-9]. These factors include scarce availability of reliable PrEP information in Spanish, limited self-efficacy on how to access PrEP care, perceived or real PrEP costs, perceived ineligibility for free or low-cost PrEP care due to having an undocumented migratory status, competing job obligations, and a limited pool of available clinics that are equipped to provide language- and culturally concordant PrEP care to Latino MSM [7-9].

Mobile health (mHealth) technology represents an innovative set of tools to increase PrEP uptake in different populations [17-22]. HealthMindr [19] is an evidence-based mobile app that has been added to the Centers for Disease Control and Prevention’s compendium of evidence-based interventions [23]. It features in-app health screenings that direct users to appropriate services: HIV testing and PrEP service locator; mail delivery of condoms, lubricant, and home HIV and sexually transmitted infection tests; and customizable HIV testing and PrEP care maintenance plans [19]. To date, no comprehensive HIV prevention app has been customized to address the PrEP accessibility barriers faced by Latino MSM, representing an important research gap.

### Objectives

The overall objective of this study was to develop and pilot-test Saludfindr, a comprehensive HIV prevention app for Latino MSM, with the ultimate goal of increasing PrEP uptake in this group. Using HealthMindr as a template app and following a modified version of the assessment, decision, adaptation, production, topical experts–integration, training, and testing framework [24], we first conducted formative work with Latino MSM to guide the adaptation process to produce Saludfindr [7]. In this paper, we present the results of the 4-month pilot study of Saludfindr, which assessed the app’s acceptability and usability.

## Methods

### Saludfindr Development

Following the assessment, decision, adaptation, production, topical experts–integration, training, and testing framework [24], we first decided to use HealthMindr as a template platform to develop Saludfindr as it focused on MSM and the outcomes of interest included PrEP initiation. We then conducted a formative assessment to guide the adaptation process. We conducted 13 in-depth interviews with Latino MSM, during which we identified the main barriers to and facilitators of PrEP uptake in this population, gauged opinions on existing HealthMindr app features, and proposed de novo Saludfindr features [7]. In addition to general PrEP uptake barriers such as low perceived PrEP eligibility, intersectional stigma, cost concerns, and PrEP misconceptions, we identified barriers that predominantly impact Latino MSM’s access to PrEP. These include scarcity of local PrEP clinics that can provide culturally and language-concordant care, limited availability of information about PrEP access in Spanish, distrust of peers as a reliable source of PrEP-related information, perceived ineligibility for low-cost PrEP services stemming from having an undocumented immigration status, anti-immigrant policies and rhetoric, and competing work obligations limiting attendance to PrEP clinic visits. As PrEP uptake facilitators, we identified that health care providers represented a trusted source of PrEP information, especially if they were familiar with prescribing PrEP and were bilingual Latino individuals themselves [7].

On the basis of these data, we decided to keep the following HealthMindr features: periodic health assessments to determine PrEP eligibility, in-app home HIV test and condom ordering to facilitate access, and frequently asked questions to increase information. We added 3 de novo Saludfindr features: customized motivational messages to incentivize users to adopt HIV prevention strategies, a customized sexual health clinic list to facilitate access, and a “Contact Us” button to provide additional information that users may need. Of note, all clinics included in the list were known to offer free or low-cost services to Latino MSM regardless of immigration status and had Spanish-language service availability. The process of determining which clinics to include is described elsewhere [7].

All app content was developed in English and Spanish. Members of our partner community-based organizations reviewed the content in both languages and provided feedback to optimize readability and cultural concordance. VDC, IB, HO, and NS developed all app content. The National Cancer Institute–designated Stephenson Cancer Center mHealth Shared Resource at the University of Oklahoma Health Sciences Center produced the app through the Insight mHealth platform.

### App Features

**Figure 1.**
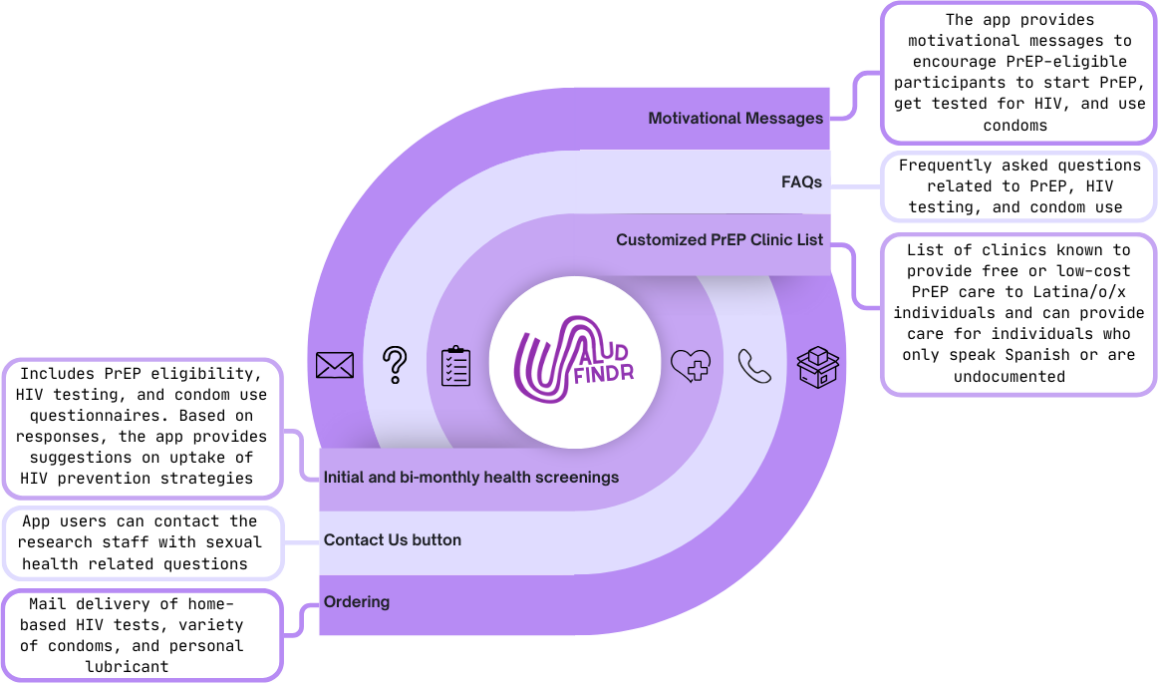
Saludfindr HIV prevention app features for Latino men who have sex with men. FAQ: frequently asked question; PReP: pre-exposure prophylaxis.

Figure 1 shows a full list of app features and descriptions.

### Pilot Study Overview

The purpose of this 4-month pilot study was to evaluate the acceptability and usability of Saludfindr among Latino MSM living in metropolitan Atlanta. Enrolled participants downloaded Saludfindr onto their Android cell phone or study-provided Android cell phone. Participants were asked the language they preferred to use the app in (English or Spanish) and interacted with it during the study period. They first completed a baseline sociodemographic survey and an initial health screening that included PrEP eligibility, HIV testing, and condom use patterns. On the basis of each individual’s responses to the initial health assessment, the app provided suggestions on PrEP use and HIV testing frequency. It also sent periodic motivational messages to encourage PrEP uptake, HIV testing, and condom use. Participants then completed 2- and 4-month health screenings that assessed needs for interim PrEP initiation and HIV testing. The 2- and 4-month health screenings also assessed the use of app features to help participants access PrEP or HIV testing services, as well as the use of the home HIV test and condom ordering feature. This was followed by customized app-based suggestions and messages. At the end of the 4-month period, participants completed a survey to evaluate the app’s acceptability and usability in assisting Latino MSM in adopting HIV prevention strategies such as PrEP uptake, HIV testing, and condom use. Throughout the study period, we gathered order tracking and app-based use data.

### Study Population, Recruitment, and Enrollment

We recruited participants from October 2022 to April 2023 using in-person and web-based recruitment strategies. All recruitment materials were available in English and Spanish. We partnered with Latino LinQ, a local community-based organization that supports, advocates for, and provides direct services to the Latina, Latino, and Latinx lesbian, gay, bisexual, transgender, and queer communities living in metropolitan Atlanta. We visited bars and clubs popular among Latino MSM with the Latino LinQ staff, who had established HIV testing stations at these venues. At these locations, trained research staff provided information about the study to attendants and distributed physical study flyers. These included a QR code that directed interested individuals to a HIPAA (Health Insurance Portability and Accountability Act)–compliant document with further information about the study and a brief eligibility survey. For web-based recruitment, Latino LinQ posted institutional review board (IRB)–approved electronic flyers in their social media accounts with a link that directed individuals to the online materials as described previously. We also conducted a social media recruitment campaign using Meta Ads Manager. Advertisements were displayed on Facebook and Instagram for 4 months. Targeted parameters included individuals aged 18 years and older residing in the Atlanta metropolitan area. Advertisements consisted of static images with a call to action directing interested individuals to the aforementioned materials.

To be eligible for this study, individuals needed to meet the following inclusion criteria (all per self-report): (1) assigned male at birth, (2) age of 18 years or older, (3) identifying as Latino, (4) having had sex with cisgender men, (5) negative or unknown HIV status, and (6) residence in the Atlanta metropolitan area.

Trained research staff (HO and IB) reviewed all electronic eligibility forms from all sources (in-person events, Latino LinQ social media, and our Meta recruitment campaign). They contacted eligible individuals, verified their identity and contact information, provided in-depth information about the study, and went over a verbal informed consent process with them over the phone. Consented participants received an email or SMS text message with instructions and a unique registration code to download the app either on their own Android cell phone or on a study-provided Android phone. Once they downloaded the app, participants completed the brief sociodemographic survey and initial health screening, which completed their enrollment into the study.

### Ethical Considerations

This study was approved by the Emory University IRB (MOD002-IRB00097743). We obtained verbal informed consent over the phone for this study. The Emory University IRB waived the requirement to obtain a written documentation of consent as this research was considered to present no more than minimal risk of harm to participants and did not involve procedures for which written consent was required. Study participants were assigned a participant identification number, and all data was deidentified prior to analysis. Participants were compensated with US $50 for the completion of the baseline sociodemographic survey and initial health screening, US $50 for the completion of the 2-month health screening, and US $50 for the completion of the 4-month health screening and app evaluation survey.

### Measures and Outcomes

In the baseline sociodemographic survey, we assessed the following: age in years, gender identity, sexual orientation, sexual partners’ gender identities, region or country of birth, immigration status, and employment status. During the initial health screening, we measured participants’ recent HIV testing history and their PrEP eligibility, which was defined as meeting at least one of the following criteria in the 6 months before enrollment: (1) engagement in condomless anal sex; (2) having had a bacterial sexually transmitted infection; (3) use of drugs such as poppers, cocaine, methamphetamines, or heroin; and (4) having had a partner living with HIV or with an unknown HIV status. During the 2- and 4-month health screenings, participants were asked whether they had started taking PrEP. Those who had started PrEP were asked whether they used the in-app customized clinic list to access PrEP care, whereas participants who had not started PrEP were asked about their reasons for not doing so, and their PrEP eligibility was reassessed. Similarly, participants were asked about interim HIV testing, test results, use of the in-app clinic list to access HIV testing services, and use of the at-home HIV test ordering app feature. Finally, we asked participants whether they ordered condoms through the app.

To evaluate the app, we assessed Saludfindr’s acceptability and usability. For acceptability, we measured the use of each app feature (from health screenings, app-based metadata, and order tracking data) and surveyed participants about the app’s acceptability in assisting Latino MSM in using HIV prevention strategies (PrEP uptake, HIV testing, and condom use). We also measured PrEP and HIV testing uptake via participant self-report through the health screenings. Usability was measured using the System Usability Scale (SUS), a validated scale that measures user ratings of product usability through 10 statements [25]. Its final score ranges from 0 to 100, with higher scores indicating higher usability. Finally, we measured uptake of HIV prevention strategies during the study period, including PrEP initiation and HIV testing.

As the main outcomes of this pilot study were acceptability and usability rather than efficacy in increasing PrEP uptake, HIV testing, or condom use, the sample size did not need to provide statistical power for detecting the app’s effects on these outcomes. A sample size of 25 to 35 participants offers enough precision to judge acceptability and usability and gather information for planning a future clinical trial [26].

A priori, we defined the following thresholds to be met by Saludfindr: (1) a 50% or higher proportion completing the 2- and 4-month health screenings; (2) a 50% or higher proportion using the at-home HIV test and condom ordering feature; (3) a 50% or higher proportion using the in-app customized clinic list to access PrEP or HIV testing services among participants who were not on PrEP at baseline or who were due for HIV testing, respectively (defined as those who had never been tested for HIV or who had been tested over 6 months before study enrollment); and (4) SUS mean score of 60/100 (good) or higher. These cutoff points informed a set of go or no-go criteria based on which Saludfindr would (1) be ready for future research during a clinical trial (all thresholds met; go), (2) need further modifications (some thresholds met; expert consultation needed), or (3) not merit further research (no go).

### Data Analysis

All analyses were conducted using the R software (R Foundation for Statistical Computing). Descriptive statistics summarized baseline characteristics, Saludfindr use, HIV prevention outcomes, perceived usefulness, and system usability. Continuous variables such as age and scores were summarized using medians and IQRs or means and SDs, as appropriate. Categorical variables were presented as frequencies and percentages. Data were analyzed from baseline sociodemographic data, in-app health assessments, the final survey, and app tracking data.

## Results

### Baseline Characteristics

We enrolled 31 Latino individuals assigned male at birth for the 4-month pilot study. The median age was 27 (IQR 24.5-32) years, 97% (30/31) were cisgender men, 81% (25/31) identified as MSM, and 19% (6/31) identified as bisexual. Approximately two-thirds (20/31, 65%) were born outside of the United States, 35% (11/31) either had an undocumented immigration status or were under humanitarian parole or asylum, and 61% (19/31) used the app in Spanish. Of all participants, 84% (26/31) had gotten tested for HIV in the previous 6 months, and 77% (24/31) were eligible for PrEP (Table 1).

**Table 1.** Baseline sociodemographic characteristics of the Saludfindr app pilot study participants (N=31).

Characteristic	Values
Age (y), median (IQR)	27 (24.5-32)
Current gender identity, n (%)	
Cisgender man	30 (97)
Sexual orientation, n (%)	
MSM^a^	25 (81)
Bisexual	6 (19)
HIV testing status, n (%)	
Within the previous 6 months	26 (84)
Over 6 months before or never tested	5 (16)
PrEP^b^ eligibility criteria met (in the previous 6 months)^c^, n (%)	
Condomless anal sex	23 (74)
Bacterial STI^d^	6 (19)
Drug use	12 (39)
Partner living with HIV or with an unknown HIV status	9 (29)
PrEP status, n (%)	
Eligible for PrEP	24 (77)
Currently on PrEP	14 (45)
Region or country of birth, n (%)	
United States	11 (35)
Mexico	8 (26)
Central America	3 (10)
South America	9 (29)
Immigration status, n (%)	
US citizen or permanent resident	17 (55)
Undocumented or on humanitarian parole or asylum	11 (35)
On visa or DACA^e^	3 (10)
Used the app in Spanish, n (%)	19 (61)

a MSM: men who have sex with men.

b PrEP: pre-exposure prophylaxis.

c PrEP eligibility was defined as meeting at least one of the following criteria in the previous 6 months: condomless anal sex, bacterial sexually transmitted infection, drug use, or having a partner living with HIV or with an unknown HIV status (participants who were found to be PrEP noneligible did not respond to the partner question and the drug use question, so we were unable to make a PrEP eligibility determination).

d STI: sexually transmitted infection.

e DACA: Deferred Action for Childhood Arrivals.

### Acceptability of Saludfindr Features

All participants completed the initial health screening, and 84% (26/31) and 77% (24/31) completed the 2- and 4-month health screenings, respectively (Table 2). Of all participants, 52% (16/31) ordered condoms through the app at least once, and 62% (10/16) of this group placed repeat orders during the pilot study. While 23% (7/31) of the participants ordered home HIV tests through the app at least once, 29% (2/7) of them placed repeat orders. Finally, 16% (5/31) of participants used the “Contact Us” button to reach out to research staff with sexual health–related questions (Table 2).

**Table 2.** Acceptability of the Saludfindr app features.

Feature	Participants, n/N (%)
Health screenings^a^	
Initial health screening	31/31 (100)
Month 2 health screening	26/31 (84)
Final health screening	24/31 (77)
Condom orders^b^	
At least once	16/31 (52)
Repeat orders	10/16 (62)
Home HIV test orders^b^	
At least once	7/31 (23)
Repeat orders	2/7 (29)
“Contact Us” button use^a^	5/31 (16)

a Data collected from app use metadata.

b Data collected from product shipping tracking.

### PrEP and HIV Testing Uptake

During the 4-month study period, 71% (22/31) of the participants got tested for HIV, 32% (7/22) of them using the in-app home HIV test ordering function and 68% (15/22) through clinic-based HIV testing. Of the 16% (5/31) of the participants who had never gotten tested for HIV or who had been tested over 6 months before study enrollment, 60% (3/5) used the in-app customized clinic list to find an HIV testing site. The remaining 40% (2/5) of the participants were lost to follow-up, so we could not document whether they got tested for HIV. At baseline, 55% (17/31) of the participants were not on PrEP. Of these, 41% (7/17) initiated PrEP during the study, and all of them did so at one of the clinics listed on the app (Table 3).

**Table 3.** Pre-exposure prophylaxis (PrEP) and HIV testing uptake among Saludfindr study participants.

Outcome	Participants, n/N (%)
HIV testing	22/31 (71)
Using in-app home HIV test ordering feature	7/22 (32)
Clinic-based testing	15/22 (68)
At clinic from in-app list	10/15 (67)
Positive HIV test	0 (0)
HIV testing among those who had never been tested or had been tested >6 months before at baseline	3/5 (60)
At one of the clinics listed on the app	3/3 (100)
PrEP initiation among those off PrEP at enrollment	7/17 (41)
At one of the clinics listed on the app	7/7 (100)

When we asked participants about Saludfindr’s usefulness in assisting Latino MSM with the uptake of HIV prevention strategies, the proportion of people who rated it as “useful” or “very useful” was 92% (22/24) for PrEP initiation, 96% (23/24) for HIV testing, and 79% (19/24) for condom use (Table 4).

**Table 4.** Perceived usefulness of the Saludfindr app for uptake of HIV prevention strategies among Latino men who have sex with men (N=24).

HIV prevention strategy	Not useful at all, n (%)	Not useful, n (%)	Neutral, n (%)	Useful, n (%)	Very useful, n (%)
PrEP^a^ uptake	1 (4)	0 (0)	1 (4)	4 (17)	18 (75)
HIV testing	0 (0)	1 (4)	0 (0)	5 (21)	18 (75)
Condom use	0 (0)	2 (8)	3 (12)	2 (8)	17 (71)

a PrEP: pre-exposure prophylaxis.

### Saludfindr Usability

Overall, Saludfindr reached an SUS score of 74.5 (excellent; Table 5). The SUS statements that obtained the highest scores included “The app was easy to use” (4.21/5), “most people would learn to use this app very quickly” (4.25/5), and “I felt very confident using the app” (4.21/5). The 2 statements with the lowest scores were “The app was unnecessarily complex” (3.12/5) and “there was too much inconsistency in this app” (3.25/5).

**Table 5.** System Usability Scale scores for the Saludfindr app during the pilot study (total calculated score: 74.5/100).

Statement	Mean (SD)^a^	Absolute^b^
I would like to use this app frequently	3.79 (1.28)	3.79 (1.28)
The app was unnecessarily complex^c^	2.88 (1.48)	3.12 (1.48)
The app was easy to use	4.21 (1.02)	4.21 (1.02)
I would need support from a technical person to be able to use this app^c^	2.46 (1.38)	3.54 (1.38)
Various functions in the app were well integrated	3.88 (1.19)	3.88 (1.19)
There was too much inconsistency in this app^c^	2.75 (1.39)	3.25 (1.39)
Most people would learn to use this app very quickly	4.25 (1.03)	4.25 (1.03)
The app was very cumbersome to use^c^	2.33 (1.43)	3.67 (1.43)
I felt very confident using the app	4.21 (1.06)	4.21 (1.06)
I had to learn many things before I could get going with this app^c^	2.67 (1.34)	3.33 (1.34)

a Scoring based on a scale from 1=“totally disagree” to 5=“totally agree.”

b Adjusts the scores of negative statements so that larger numbers are associated with positive statements.

c Negative statements.

## Discussion

### Principal Findings

In this pilot study assessing the acceptability and usability of an HIV prevention app tailored for Latino MSM living in metropolitan Atlanta, most enrolled participants were young, non–US born, primarily Spanish speakers, and eligible for PrEP. The intervention, Saludfindr, met or surpassed prespecified targets for acceptability and usability, including the percentage of health screenings completed, in-app condom ordering, use of in-app customized clinic lists to access PrEP services among participants off PrEP at baseline, and HIV testing among participants due for it. It also surpassed the predetermined SUS score. Saludfindr did not meet the target for use of in-app home HIV test ordering. This indicates that further modifications to this feature are needed to address potential user-related uptake barriers. Taken together, these findings indicate that a clinical trial to determine the app’s efficacy to promote PrEP uptake, HIV testing, and condom use is merited.

Compared with the HealthMindr pilot study, Saludfindr had a higher percentage of PrEP uptake among PrEP-naive users (7/17, 41% vs 9% during the former study [19]). PrEP use at baseline was also higher among Saludfindr participants than among HealthMindr participants (14/31, 45% vs 12%, respectively). These differences could be explained at least in part by the 6-year gap between the HealthMindr and Saludfindr pilot studies. Between 2017, when the HealthMindr pilot study was conducted, and 2023, PrEP awareness and initiation and willingness to use it among MSM increased overall across races and ethnicities in the United States [16,27,28]. In addition, all Saludfindr participants who started PrEP during the study did so at one of the clinics we listed on the app. This new feature to highlight clinics friendly to Latino MSM was not available in the original HealthMindr study, suggesting the importance of this newly added feature. Further facilitating PrEP access for Latino MSM through the app may be a more focused area of intervention to increase PrEP uptake in this group. For example, a future study could combine the customized clinic list feature with other evidence-based support such as PrEP navigation assistance [29,30].

In terms of HIV testing, the proportion of Saludfindr participants who ordered a home HIV test through the app was lower than that of HealthMindr participants and lower than those of other interventions for home HIV testing among Latino men, where uptake of home HIV testing ranged between 30% and 70% [31-34]. This finding may be due in part to the high uptake of PrEP, which entails regular HIV testing and, as such, additional home testing would not be needed. Among Saludfindr participants who did use HIV testing, most HIV tests were conducted at a clinic, often one of the clinics listed on the app. Our research staff reported that, during conversations about the app and its functionalities, some participants voiced hesitancy in ordering home HIV tests out of concerns about making mistakes while getting tested, which could lead to inaccurate test results. They were also concerned about interpreting test results correctly and what to do if they had a positive result. These results and insights suggest that, for future Saludfindr versions, additional in-app content may need to be added to increase users’ self-efficacy in using home HIV tests.

Saludfindr reached comparable SUS scores to those of HealthMindr (74.5 vs 73, respectively) and DOT Diary (SUS score of 76.4), a mobile app focused on PrEP retention in care and adherence and adapted for Latino MSM and transgender women’s needs [35]. To optimize usability, future Saludfindr versions will need to include automatic data upload from the app to the cloud. The study version required users to tap the “Sync” button every time they used the app, which may explain why some participants found the app “unnecessarily complex.”

### Limitations

Our study had several limitations. First, Saludfindr’s software was only compatible with Android phones. Even though we provided study cell phones to participants who did not have an Android phone, it represented a barrier to how seamlessly we could enroll participants into the study. Second, similar to all prevention studies, there was a risk of selection bias because individuals more familiar with prevention modalities may have been more motivated to participate in the study. Third, we only enrolled participants living in the Atlanta area as our formative work to develop the app and its features was based in this area. As such, our study results do not necessarily generalize to Latino MSM living in other areas of the country, including those living in rural areas or in states with enhanced support services for this population. Fourth, our sample size was relatively small compared to other mobile app–based studies. That said, we were able to enroll a population with multiple barriers to accessing PrEP and historically identified as “hard to reach.” As such, their specific feedback related to an HIV prevention app is extremely valuable to optimize Saludfindr and guide other investigators working on digital HIV interventions for this group.

### Conclusions

Despite these limitations, Saludfindr represents the first bilingual mobile app aiming to increase PrEP uptake, HIV testing, and condom use that has been tailored to the needs of primarily Spanish-speaking Latino MSM in the United States. There has been some movement to develop adherence support apps for Latino MSM, such as DOT Diary by Liu et al [35], a mobile app to support PrEP retention in care and adherence by combining electronic pill taking technology and a sex diary to provide PrEP protection-level feedback to users. Liu et al [35] adapted and piloted DOT Diary to meet the needs of Spanish-speaking MSM and transgender women in San Francisco and Miami, attaining high SUS scores, and it is currently being assessed for effectiveness in the setting of a clinical trial.

Overall, Saludfindr represents an innovative, culturally responsive tool that, once optimized, could contribute to efforts to curb the HIV epidemic among Latino MSM in the United States. Saludfindr, if proven effective in increasing PrEP uptake, HIV testing, and condom use among Latino MSM in a future clinical trial, could be deployed by health departments in other southern US cities and rural areas to assist in narrowing the existing racial and ethnic gap in PrEP uptake. We believe that this would be feasible as the app was well accepted by future users and it is easy to install and use. As with any tool, its effectiveness in decreasing HIV incidence in this population will likely be enhanced through combination with other strategies, mHealth-based or of other nature, as barriers to accessibility to HIV prevention services among this group are complex and multifactorial.

## Abbreviations

HIPAA: Health Insurance Portability and Accountability Act
IRB: institutional review board
mHealth: mobile health
MSM: men who have sex with men
PnR: pre-exposure prophylaxis–to-need ratio
PrEP: pre-exposure prophylaxis
SUS: System Usability Scale
